# Ion release and pH of a new endodontic cement, MTA and Portland cement

**Published:** 2009-04-17

**Authors:** Sara Amini Ghazvini, Maryam Abdo Tabrizi, Farzad Kobarfard, Alireza Akbarzadeh Baghban, Saeed Asgary

**Affiliations:** 1 *Researcher, Dental Research Center, Dental School, Shahid Beheshti University of Medical Sciences, Tehran, Iran.*; 2 *Department of Operative Dentistry, Dental School, Shahid Beheshti University of Medical Sciences, Tehran, Iran.*; 3 *Department of Medicinal Chemistry, School of Pharmacy, Shahid Beheshti University of Medical Sciences, Tehran, Iran.*; 4 *Department of Biostatistics, Paramedical School, Shahid Beheshti University of Medical Sciences, Tehran, Iran.*; 5 *Department of Endodontics, Iranian Center for Endodontic Research, Dental Research Center, Dental School, Shahid Beheshti University of Medical Sciences, Tehran, Iran.*

**Keywords:** Calcium, CEM cement, Ion release, MTA, NEC, New material, pH, Phosphate, Portland cement

## Abstract

**INTRODUCTION:** This *in vitro* study measured and compared pH and phosphate and calcium ions release of a new endodontic material (CEM cement), mineral trioxide aggregate (MTA), and Portland cement (PC) using UV-visible technique, atomic absorption spectrophotometry methods, and pH meter, respectively.

**MATERIALS AND METHODS:** Each material was placed in a plastic tube (n=10) and immersed in a glass flask containing deionized water. Half of the samples were tested for determining pH and released ions after 1h, 3h, 24h, 48h, 7d and 28d. Remaining samples (n=5), were evaluated after 28d. Data was analyzed using one way ANOVA and Tukey tests.

**RESULTS:** Results indicated that all materials were highly alkaline and released calcium and low concentration of phosphate ions in all the time intervals. CEM cement released considerably higher concentration of phosphate during the first hour (P<0.05).

**CONCLUSION:** This novel endodontic cement promoted alkaline pH in a similar manner to MTA and released calcium and phosphate. These conditions can stimulate the calcification process and explain the basic physico-chemical mechanisms of hard tissue regeneration of CEM cement.

## INTRODUCTION

Mineral trioxide aggregate’s (MTA) chief ingredient is Portland cement (PC) (1). MTA has been recommended for vital pulp therapy, root-end filling, apexification, and perforation repairs (2). Research studies have demonstrated comparable physical, chemical and biological properties for gray and white MTA with regular and white PC (3-7).

Recently, new endodontic cement (NEC) in the name of calcium enriched mixture (CEM) cement with a different chemical composition from MTA (8) but the same clinical applications has been developed (9-10). CEM cement is composed of different calcium compounds i.e. calcium hydroxide, calcium oxide, calcium phosphate, calcium sulfate, calcium silicate, and calcium carbonate (9). Although *in vitro* sealing ability (9,11) and *in vivo* vital pulp therapies of CEM cement and MTA revealed similar results (10,12), CEM cement offers some benefits over MTA such as improved handling, shorter setting time, more flow and less film thickness (8), ability to form hydroxyapatite in normal saline solution (13), as well as an estimated lower cost. Research suggests that the high pH and released calcium and phosphorus ions are required for a material to stimulate mineralization in the process of hard tissue healing (14). The excellent biocompatibility of MTA, hydroxyapatite and other calcium-containing materials may contribute to their ability to release calcium ions which react with phosphate ions of body tissue fluid, resulting in hard tissue formation. Sarkar *et al.* reported that MTA in synthetic tissue fluid produced precipitates with similar composition and structure to hydroxyapatite (15). Furthermore, increasing pH levels contributed to antibacterial activity; a critical factor in the formation of a mineralized tissue barrier (16). Despite the importance of phosphate ion presence in hydroxyapatite formation (15), literature review has not revealed research that discussed phosphate ion release from MTA or PC. The calcium ions released and the pH of MTA has investigated, however no comparisons to PC have been made (17-18).

Moreover, in some previous studies (17-18) the early setting time; the most important period for ion release and structure formation (19), has been ignored. Also hard tissues formation requires over 4 weeks; and the hydration reaction of PC is a continuous process requiring longer periods of evaluation (20).

Therefore, the aim of this *in vitro* study was to measure and compare the pH and calcium and phosphate ion release of CEM cement with those of ProRoot MTA and PC, both periodically and cumulatively during 28 days.

## MATERIALS AND METHODS

The evaluated materials in this *in vitro* study were ProRoot MTA (Tooth-colored, Dentsply, Tulsa Dental, Tulsa, Ok, USA), CEM cement and PC (Abiek cement, Qazvin, Iran). MTA, CEM cement and PC were mixed with the solutions provided by the manufacturer, inventor, and deionized water, respectively. The cements were inserted into plastic tubes 10 mm long and 1.5 mm in diameter which were prewashed with 5% nitric acid to prevent interference with phosphate ion and alkaline metals. The tubes were weighed before and after filling.

A total of 10 samples were chosen for each material. Five empty plastic tubes were used as the negative control. Each specimen was immediately immersed in a flask containing 10 mL of deionized distilled water; sealed and stored at 37ºC and a relative humidity of >90%. Evaluations were performed at periods of 1h, 3h, 24h, 48h, 7 days and 28 days after immersion. Following each measurement, half the specimens (n=5) were removed carefully and placed in another flask with an equal amount of fresh deionized water. Remaining samples (n=5) stayed sealed in the bath for 28 days.

***Calcium ion analysis***

An atomic absorption spectrophotometer (Perkin-Elmer model 1100B, Phoenix, Arizona, USA) with a flame mode was used to measure calcium ion release under the following operating conditions ; 1) lamp: calcium, 2) fuel: acetylene and 3) oxidant: air.

Standard solutions containing calcium concentrations of 1, 2, 3, 4 and 5 ppm were used to create a standard calibration curve. One hundred µL of each sample was diluted with 900 µL of deionized distilled water. Twenty µL of 5% lanthanum chloride was added to eliminate interference; solutions were then injected into atomic absorption specrophotometer. The results were calculated for the above samples with a ×10 correction factor.

*Phosphate ion analysis*

UV-visible spectrophotometer (Shimadzu 160A UV-visible, Kyoto, Japan) was adjusted at wavelength of 650 nm to evaluate the phosphate ion released.

Phosphate ion concentration was determined using a photometric method. In brief, a standard curve was constructed using standard phosphate concentrations of 0.05, 0.1, 0.2, 0.3, 0.4 and 0.5 ppm in deionized water with UV absorption of 0.01, 0.027, 0.051, 0.072, 0.096 and 0.126, respectively. Twenty microliters of acidic solution of ammonium heptamolibdate was added to 5 mL of the sample in a test tube. After adding two drops of stannous chloride in glycerin as a reducing agent, the mixture was shaken. Molibdo-phosphoric acid forms in the presence of phosphate, a compound which can be reduced to a blue complex. Readings were taken 10±1 minutes after mixing.

***pH analysis***

For measurement of pH a Metrohm 744 pH meter (Metrohm Ltd, Herisau, Switzerland), calibrated with buffer solutions at pH 4.0 and 7.0, at 29**º**C was used.

**Table 1:** Analysis of calcium ion release values (Mean±SD) recorded at different time intervals ^a^

**Time **	**1h**	**3h**	**24 h**	**48 h**	**7 d**	**28 d**
**WMTA**	^a^35.29^5^ (2.29)	^a^9.80^1^ (1.93)	^a^14.40^12^(6.65)	^a^19.20^23^(2.16)	^a^25.00^34^(5.67)	^a^30.20^45^(2.78)
**CEM cement**	^a^35.40^2^ (23.2)	^b^31.20^2^ (3.96)	^b^33.60^2^(5.41)	^b^6.40^1^(4.50)	^a^28.40^2^(5.32)	^a^38.20^2^(10.0)
**PC**	^a^23.00 (6.24)	^c^24.80 (4.21)	^b^29.60(6.27)	^c^28.20(3.56)	^a^29.20(3.97)	^a^31.80(7.53)

*a:Values followed by different numbers (1-5) demonstrate statistically significant differences for calcium ion release within a material at different times; different letters (a, b, c) show statistically significant differences for calcium ion release in study groups at a particular time*

**Table 2:** Analysis of Phosphate ion release values (Mean±SD) recorded at different time intervals ^a^

**Time **	**1h**	**3h**	**24 h**	**48 h**	**7 d**	**28 d**
**WMTA**	^a^0.21^1^ (0.08)	^a^0.49^1^(0.31)	^a^0.16^1^(0.11)	^a^0.29^1^(0.12)	^a^0.33^1^(0.18)	^a^0.29^1^(0.24)
**CEM cement**	^b^0.73^2^(0.50)	^a^0.47^12^(0.26)	^a^0.26^12^(0.12)	^b^0.11^1^(0.05)	^a^0.17^1^(0.08)	^a^0.16^1^(0.05)
**PC**	^a^0.10^1^(0.03)	^a^0.17^12^(0.03)	^a^0.23^123^(0.08)	^ab^0.26^23^(0.09)	^a^0.34^3^(0.12)	^a^0.10^1^(0.02)

*a: Values followed by different numbers (1-3) demonstrate statistically significant differences for phosphate ion release within a material at different times; different letters (a, b, c) show statistically significant differences for phosphate ion release in study groups at a particular time*

**Table 3:** Analysis of pH values (Mean±SD) recorded at different time intervals ^a^

**Time **	**1h**	**3h**	**24 h**	**48 h**	**7 d**	**28 d**
**WMTA**	^a^10.61^12^ (0.63)	^a^9.63^3^(0.30)	^a^10.28^12^(0.08)	^a^10.09^23^(0.28)	^a^10.59^12^(0.18)	^a^10.80^1^(0.08)
**CEM cement**	^a^10.71^1^(0.24)	^a^10.02^3^(0.24)	^a^10.41^12^(0.14)	^a^10.16^23^(0.11)	^a^10.43^12^(0.15)	^b^10.65^1^(0.05)
**PC**	^a^10.00^1^(0.56)	^a^9.71^1^(0.18)	^b^9.92^1^(0.26)	^a^9.47^1^(0.76)	^a^9.89^1^(0.75)	^b^10.54^1^(0.10)

*a: Values followed by different numbers (1-3) demonstrate statistically significant differences for pH within a material at different times; different letters (a, b) show statistically significant differences for pH in study groups at a particular time*

***Statistical analysis***

Type I error was assumed as α=0.05. The results from the dependent variable i.e. pH, calcium and phosphate release at the various time intervals were analyzed using one-way and two-way ANOVA. Tukey test was performed for multiple comparisons. One-way ANOVA was used to compare the materials’ cumulative data.

## RESULTS

Negative controls displayed a neutral pH and lacked ion released. Table 1, Table 2, and Table 3 present mean values of calcium and phosphate ion released and pH in tested cements, respectively. There is significant difference in concentration of calcium ion released from MTA (P<0.001) and CEM cement (P<0.01) at the various time intervals. PC did not show a significant difference. CEM cement demonstrated the highest calcium ion release in the first 24 hour of immersion with the minimal changes. Also when considering calcium ion release, a constant balance is presumably quickly reached between PC and the aqueous media.

All samples released low concentrations of phosphorous; CEM cement however, produced significantly more phosphate ions than the others during the first hour (P<0.05) (Table 2).

MTA, CEM cement, and PC generated an alkaline pH of 9.47-10.80 (Table 3). The cements did not show significant difference in pH at the various time intervals except for 24h and 28 days. Contrary to MTA and CEM cement (P<0.001), PC produced relatively constant pH throughout the experiment. The highest pH was observed after 1 hour and 28 days in all groups. In the cumulative analysis, after 28 days of study, the materials showed no significant difference in either pH or calcium and phosphate ion release.

## DISCUSSION

The close proximity of media to the oral tissue fluid (containing calcium and/or phosphate) maybe a source of calcium phosphate nucleation (13); hence deionized distilled water was chosen as the storage media.

It is widely known that MTA originates from Portland cement. PC mainly consists of tricalcium silicate (C3S), dicalcium silicate (C2S), tricalcium aluminate, and tetracalcium aluminoferrite, and when mixed with water, generates calcium hydroxide (CH) which ionizes and releases calcium and hydroxyl ions (20). The complicated hydration reaction of PC mainly consists of C3S and C2S compounds:

**2[3CaO.SiO**_2_ **]+7H**_2_ **O→3CaO.2SiO**_2_ **.4H**_2_ **O+3Ca(OH)**_2_

**2[2CaO.SiO**_2_ **]+5H**_2_ **O→3CaO.2SiO**_2_ **.4H**_2_ **O+Ca(OH)**_2_

While C3S sets much faster, C2S provides physical strength after one week (20). As demonstrated above production of CH in the hydration reaction of C3S is more than C2S. The rise in pH and the calcium ion released are two major consequences of CH ionization (21); this may explain the higher pH and calcium ion released during the initial 1h period, concurring with Popovic’s results (22).

After 28 days pH remains high indicating continual reaction and setting of tested material concurring with other studies (23).

Calcium hydroxide, a main by-product of PC, MTA, and CEM cement, has a pH of 12.5 (8,22,24). The antibacterial characteristic of CH is attributed to the release of hydroxyl ions (25). While a pH greater than 9 may reversibly or irreversibly inactivate bacterial cellular membrane enzymes resulting in a loss of biological activity (26), a pH greater than11.5 is inhibitory for majority of bacteria specially *Enterococcus faecalis* (16). Although all tested materials showed pH more than 9, a recent study revealed that antibacterial activity of CEM cement is comparable with CH and significantly greater than MTA (27). It has been hypothesized that CEM cement contains greater potent antibacterial inhibitors than MTA.

An alkaline environment is a key factor that assists in the healing of pulp tissue and mineralization (28). Our results demonstrated that MTA, CEM cement and PC all produced an alkaline pH of ~9.5-11, agreeing with a previous study (8); however, Torabinejad *et al.* reported values higher than 12.0 for MTA (24). Their pH measurements were dissimilar; pH was directly measured from the cement mass using electrodes rather than immersion samples in deionized distilled water. Our technique has the advantage of allowing measurements at periods longer than the working time; therefore representing the cement’s ability to increase pH. As high pH accelerates hydroxyapatite formation (14,29) and decreases calcium phosphate solubility (18), the favorable results of *in vivo* studies for hard tissue formation becomes evident (6,7,10,12).

Interestingly the cements released phosphate ions in low concentrations. Except for CEM cement at the first hour, concentration of phosphate ions released from cements was not statistically different amongst cements. Previous studies showed trace amounts of phosphorus in MTA and PC compositions (4,30,31), however, a recent study showed that CEM cement contains significantly higher concentration of phosphorus than MTA (8). CEM cement had the ability of hydroxyapatite formation in normal saline solution from the indigenous sources (13). Therefore, despite the presence of high concentration of phosphorous in CEM cement, it seems reasonable to suspect that the presence of low concentration of phosphate ions in CEM cement media is probably due to its reaction with released calcium ion to form hydroxyapatite in the first hour.

## CONCLUSION

Within the limitations of this *in vitro* study, the results suggest that tested materials released large amounts of calcium ion, small amounts of phosphate ions, and promoted an alkaline pH, explaining CEM cement, MTA and PC’s favorable biocompatibility.
